# A Quasi-Experimental Study Investigating the Impact of a Lifestyle Redesign Program on the Well-Being of Korean University Students

**DOI:** 10.1155/2024/2683453

**Published:** 2024-02-15

**Authors:** Sun-Joung Leigh An, Gyu-Rin Kim

**Affiliations:** ^1^Department of Occupational Therapy, Graduate School of Inje University, Gimhae-si, Republic of Korea; ^2^Department of Rehabilitation Science, Graduate School of Inje University, Gimhae-si, Republic of Korea; ^3^Ubiquitous Healthcare Research Center, Inje University, 197 Inje Rd, Gimhae-si, Gyeongsangnam-do 621-749, Republic of Korea; ^4^HOPE Parent Training Center, Seoul, Republic of Korea

## Abstract

**Background:**

Korean university students (KUS) face numerous challenges that can jeopardize their well-being, including academic stress, peer pressure, irregular sleep patterns, unhealthy eating habits, lack of physical exercise, and difficulties in time management, resulting in unhealthy habits and fluctuations in lifestyle. Consequently, there is a growing need for interventions tailored to this population.

**Aim:**

This study explored the effects of a Lifestyle Redesign (LR) intervention on Korean university students' well-being including occupational participation, satisfaction, perceived stress levels, and quality of life.

**Method:**

A quasi-experimental study with 33 KUS (17 intervention, 16 control) assessed the effects of a 10-week LR intervention on well-being of the students. Pre- and postintervention changes were measured using Canadian Occupational Performance Measure (COPM), Stress Response Inventory (SRI), and World Health Organization Quality of Life Scale Abbreviated Version (WHOQOL-BREF). The intervention, delivered by trained OTs, comprised of individual and group sessions.

**Results:**

Statistically significant improvement was observed in occupational performance. While statistical significance was not consistently achieved in the rest of other areas, the LR group displayed positive trends. The LR group exhibited higher COPM satisfaction scores, lower SRI scores (indicating reduced stress), and elevated WHOQOL-BREF scores compared to the control group.

**Conclusion:**

This study contributes to the understanding of the importance of addressing lifestyle changes and habits in the well-being of university students, especially in the context of academic stress and peer pressure. Future research with larger, more diverse samples and extended intervention periods may offer further insights into the benefits of LR programs in university settings.

## 1. Introduction

The well-being and mental health of university students have become global concerns in recent years, particularly during the critical transition from high school to university marked by newfound independence, academic pressures, and lifestyle changes [1, 2]. Students worldwide are faced with new life experiences when enrolled at a university, including heightened exposure to various health risk factors such as increased alcohol consumption, tobacco use, and risky sexual behaviors [3, 4]. These factors collectively contribute to a crucial phase in which students establish habits that can significantly impact their physical and mental health throughout their lives.

Globally, university students encounter common challenges during this transition, including disruptions to established habits, challenges in time management, academic pressures, irregular sleep patterns, forging new social connections, and adapting to unfamiliar surroundings [5]. Consequently, mental health issues such as anxiety and depression are prevalent among university students worldwide due to these common challenges [3, 6]. Additionally, maintaining a healthy lifestyle during these critical years is a significant global concern. Research has shown that a substantial proportion of students experience weight gain during their first year at a university due to stress, unhealthy dietary choices, and decreased physical exercise [5, 7, 8]. The pervasive use of digital devices, such as cell phones and computers, contributes to a sedentary lifestyle, reducing motivation for physical activities and increasing the risk of weight gain [5].

In comparison to their global counterparts, Korean university students (KUS) are faced with the distinctive challenge of an intensely competitive university entrance exam. The university entrance exam in Korea is renowned for its competitiveness, and students who do not perform well may face difficulties in gaining admission to their preferred universities [9]. This intense academic environment contributes significantly to stress and anxiety among KUS. The pressure to excel in the highly competitive entrance exam creates an atmosphere where mental health concerns are heightened. Moreover, the substantial time invested in preparing for the exam may impede the development of individual identities among KUS, potentially delaying their self-discovery and identity formation [1].

While university students worldwide encounter common challenges in adapting to new environments and managing stress, the unique stressors and cultural factors faced by KUS warrant particular attention. South Korea's emphasis on high academic achievement, social conformity, and patriarchal Confucian norms can exacerbate stress levels and hinder students' ability to cope with challenges effectively. KUS face significant social pressure to excel academically, secure stable employment, enter into successful marriages, and start families, imposing a substantial burden on young individuals.

Cultural factors play a pivotal role in influencing the mental health of KUS. Korean culture places a strong emphasis on collectivism and conformity, which can constrain individuality and self-expression and intensify peer pressure. Moreover, the prevailing patriarchal Confucian culture in Korea acts as an additional hindrance to students' ability to mold their identities and make independent decisions [1]. Rooted in the Confucian emphasis on hierarchy, obedience to authority figures—be it parents or educators—and a perceived obligation to prioritize others' needs, this dynamic contributes to burnout and heightened stress levels.

The high prevalence of unhappiness and mental health issues among KUS is a pressing concern that necessitates attention and intervention to enhance students' overall health and well-being. A study conducted by Lee and Padilla [9] discovered that South Korea ranked the lowest in terms of happiness among 30 other industrialized countries globally. The study identified that students' lower happiness scores were correlated with stressors like academic performance, school-related violence, cyberbullying, Internet addiction, and unhealthy lifestyles.

A healthy lifestyle is characterized by consistent physical activity, a nutritious diet, and a well-regulated sleep schedule. However, transitioning to a new environment can disrupt established habits, leading to significant changes in one's way of life. Common unhealthy habits among KUS include skipping breakfast, irregular meal patterns, infrequent exercise, and erratic mealtimes [10]. These habits can exacerbate stress levels and hinder effective coping with challenging emotions, potentially contributing to mental health concerns.

In response to increasing mental health challenges of university students, particularly those facing unique cultural and academic pressures like KUS, various interventions have emerged, including counseling, cognitive behavioral therapy, mindfulness programs, educational programs tailored to mental health, and pharmacological interventions. However, these approaches often primarily focus on symptom management and cognitive restructuring [8], neglecting the potential of addressing lifestyle factors and occupational participation.

Lifestyle Redesign (LR) presents itself as a holistic and promising intervention for KUS. Grounded in the principles of occupational science [11], LR empowers individuals to develop healthy routines, cultivate beneficial habits, and engage in meaningful activities, leading to improved well-being [12–14]. This multifaceted program incorporates client education, occupational analysis, problem-solving, motivation enhancement, and strategies for lasting behavioral change [15, 16].

LR's unique focus on lifestyle and occupational participation [12–14] positions it as a promising tool in addressing the challenges faced by KUS outlined earlier, such as intense academic pressure and cultural expectations. Through LR, KUS can develop healthy coping mechanisms, effectively manage stress, and ultimately achieve improved overall well-being, potentially going beyond symptom management and cognitive restructuring often associated with existing interventions.

This study is aimed at understanding how LR, a preventive intervention method in occupational therapy, can address these specific challenges in the Korean context.

The objectives of this research are as follows:

1. To investigate the effects of the Lifestyle Redesign program intervention on the occupational participation of KUS measured by the occupational performance and satisfaction
2. To investigate the effects of the Lifestyle Redesign program intervention on the well-being of KUS based on the perceived stress level and quality of life

This research is aimed at contributing valuable insights into the potential benefits of Lifestyle Redesign as an intervention to promote the well-being of KUS facing unique challenges in their academic journey.

## 2. Method

### 2.1. Study Design

A quasi-experimental design with two group pre-post comparison was used. The two groups were Lifestyle Redesign (LR) intervention group and a control group. Ethical approval was granted by the IJ University Institutional Review Board. A convenience sample of students enrolled in occupational therapy at IJ University in Korea was recruited.

### 2.2. Recruitment

At the start of the semester, the research team made a recruitment announcement to all of the occupational therapy students at IJ University about the research. Those who were interested and volunteered to participate in this study were recruited.

### 2.3. Participants

33 students participated in this study. The study initially started with a total of 40 students; however, 3 students chose to discontinue their involvement during the course of the study. Additionally, 4 students who did not provide complete responses to all survey questions were subsequently excluded from the study.

The inclusion criteria for the study were as follows:

1. Enrolled as a student in Occupational Therapy at IJ University at the time of this study
2. Demonstrated the ability to comprehend and adhere to the instructions provided by the researcher
3. Providing explicit written consent to partake in the study

The exclusion criteria for the study were as follows:

1. Did not complete the tests or questionnaires
2. Did not provide written consent to partake in the study

### 2.4. Procedures

The study began with individually explaining its purpose and procedures to each participant and obtaining their informed consent. Subsequently, participants were randomly assigned to either the LR intervention group or the control group through a random drawing process.

Before the intervention began, all participants, regardless of their group assignment, completed a battery of self-reported pretest assessments. These assessments included the Canadian Occupational Performance Measure (COPM), the Stress Response Inventory (SRI), and the World Health Organization Quality of Life Scale Abbreviated Version (WHOQOL-BREF). Additionally, participants in the LR intervention group also filled out an occupational questionnaire aimed at understanding their daily activities.

Following the pretest, all participants attended a lecture emphasizing the importance and necessity of engaging in meaningful activities. Over the following 10 weeks, the LR intervention group received tailored LR interventions, guided by six occupational therapists (OTs) trained in LR. Throughout the 10-week intervention period, participants in the control group received weekly check-in calls and personalized emails from the research team. These interactions served as a means of providing verbal and written encouragement to participants to maintain a regular routine of three meals daily and at least 30 minutes of exercise three times per week.

**Figure 1:**
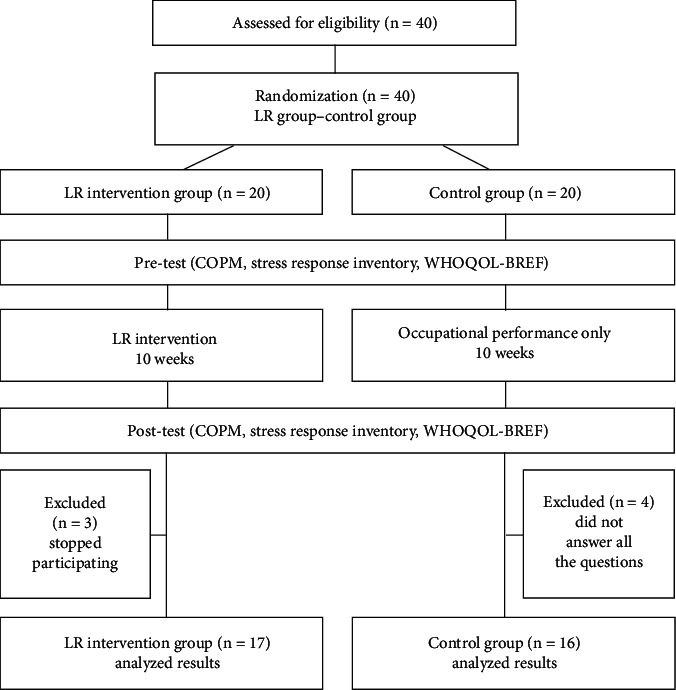
Flow diagram of the research process.

Following the 10-week intervention period, both the LR intervention group and the control group completed the same battery of self-reported assessments used in the pretest (see Figure 1). This allowed researchers to compare pre- and postintervention scores and assess the effectiveness of the LR intervention on participants' occupational performance, stress response, and quality of life.

### 2.5. Lifestyle Redesign Intervention

Grounded in the theoretical framework of Mandel et al. [14], the LR intervention adopted a client-centered approach, tailoring its components to address the unique needs of each participant. The primary focus of this intervention has centered on effectively managing and planning the utilization of time and activity patterns, promoting the adoption of healthy habits, and encouraging meaningful activity engagement, all aimed at enhancing the overall quality of life for the participants [13].

Both the LR intervention and control groups participated in a preliminary lecture highlighting the importance of engaging in meaningful activities for overall health and well-being. This is aimed at providing a shared foundation of understanding and encouraging participants to consider the potential benefits of such activities in their own lives (Clark et al. [13]).

Additionally, both the LR intervention and control groups shared certain components in the intervention program. These included the practice of consuming three meals daily, engaging in at least 30 minutes of exercise three times a week, involvement in meaningful activities, and stress management.

During this intervention phase, the control group received verbal and written encouragement only. In contrast, the LR intervention group experienced a more structured approach. Participants in this group were organized into smaller groups of 3-4 students, led by an OT trained in LR. Each group underwent the LR intervention, fostering a more personalized and interactive experience.

Participants in the LR intervention group subsequently completed an occupational questionnaire (OQ), enabling them to document their daily activities. The six OTs then conducted individual 1 : 1 interviews with each participant, during which they reviewed and analyzed the OQ. In these interviews, the OTs collaborated with the participants to identify opportune times for integrating meals and exercise into their daily routines. Additionally, the OTs assisted participants in identifying meaningful activities that aligned with their personal preferences and desires. Furthermore, the individual interviews provided a platform for the OTs to assess each participant's specific needs, taking into account factors such as their environmental context and financial situation. This information was then employed to guide participants in selecting appropriate and meaningful activities.

To ensure that participants in the LR intervention group continued to actively engage in meaningful activities, the OTs conducted individual 30-minute sessions with each participant once a week. These weekly meetings served as opportunities to pinpoint and address any barriers or challenges that participants encountered in their pursuit of these activities. In addition to these individual sessions, participants attended in 1-hour small group sessions once every week. The topics in the group sessions included information about power of occupation, time management, healthy lifestyle, stress management, healthy eating habits, and physical activity.

The group activities and discussions were intentionally structured to empower participants, enabling them to boost their overall well-being through the adoption of health-promoting activity choices (see Table 1). The group sessions included short lectures on the topics followed by group discussions and personal exploration.

For instance, participants collectively assessed their daily routines and engaged in discussions to identify opportunities for incorporating exercise or implementing stress management techniques as deemed suitable for their individual circumstances. Within these group meetings, participants not only discussed these practical aspects but also shared their personal experiences. Together, they collaboratively crafted narratives that revolved around their engagement in various activities. This process was thoughtfully designed to facilitate participants in the exploration of their own identities and to reinforce the significance of actively participating in meaningful activities in their lives.

### 2.6. Outcome Measures

#### 2.6.1. Canadian Occupational Performance Measure (COPM)

The COPM is a client-centered tool designed to assess perceived occupational performance and satisfaction over time [17]. The participant is able to self-identify areas in occupational performance they want to work on and assign scores on a scale of 1 to 10, with higher scores signifying greater performance and satisfaction levels. COPM is recognized for its reliability, validity, and responsiveness in measuring performance change linked to specific goals. According to Law et al. [17], a difference of 2 or more points between the average pre- and postintervention COPM scores indicates notable clinical significance.

#### 2.6.2. Stress Response Inventory (SRI)

Koh et al. [18] introduced the SRI. Comprising a total of 39 questions, this inventory is structured to assess stress response across emotional, physical, cognitive, and behavioral dimensions. Each question is rated on a 5-point Likert scale, with a higher cumulative score indicating elevated stress levels.

#### 2.6.3. World Health Organization Quality of Life Scale Abbreviated Version (WHOQOL-BREF)

The overall health and quality of life were measured by the Korean version of the WHOQOL-BREF, which was developed by Min et al. [19]. There are 4 domains: physical health, psychological health, social relationships, and environment. Each question within these domains is assigned a score on a Likert scale. A greater score corresponds to a higher quality of life.

#### 2.6.4. Occupational Questionnaire

The occupational questionnaire (OQ) was developed by Smith et al. to assess volition subsystems and activity patterns [20]. To successfully complete the OQ, participants specify their primary activity for each half-hour period during a regular day when they are awake. They categorize each activity as either work, daily living task, recreation, or rest. In the context of this study, a modified version of the OQ was utilized to investigate how participants allocated their time and engaged in various occupations.

### 2.7. Data Analysis

The data were analyzed using the statistical software package program IBM SPSS Statistics version 18.0. Descriptive statistics were applied to the participants' demographic information. An independent samples *t*-test was employed to compare the differences between the two groups preintervention, at baseline. To assess changes within each group pre- to postintervention, the Wilcoxon signed-rank test was utilized. The Mann–Whitney *U* test was employed to analyze the changes from pre- to postintervention between the two groups. The significance level was set at *p* < 0.05.

## 3. Results

A total of 33 students, 17 in LR intervention group and 16 in control group, participated in this study. There were 2 (11.8%) male and 15 (88.2%) female students in the LR intervention group and 3 (18.8%) male and 13 (81.3%) female students in the control group. The mean age of the intervention group was 20.10 years, and the mean age of the control group was 20.0 years. General characteristics of the students are provided in Table 2. No significant differences were observed between the two groups in the outcome measures of the preintervention phase. This indicates that the initial values of the two groups were similar.

The analysis of pre- and postintervention scores within the intervention group revealed noteworthy improvements in both COPM performance and satisfaction scores. Statistically significant distinctions were evident in performance (*p* = 0.006) and satisfaction (*p* ≤ 0.001), as shown in Table 3. These changes were statistically significant, indicating that the LR intervention effectively improved both performance and satisfaction in daily activities among participants.

The SRI scores indicated a statistically significant reduction in reported stress levels, decreasing from 67.88 ± 21.29 to 59.06 ± 20.26 (*p* ≤ 0.001, Table 3), indicating a decrease in reported stress levels following the intervention. Notably, improvements were also observed across all quality-of-life categories of WHOQOL-BREF, with statistically significant increases in physical health (*p* < 0.05), psychological health (*p* < 0.05), environment (*p* < 0.05), and total scores (*p* < 0.05). However, the improvement in social relationships did not reach statistical significance (*p* > 0.05). This suggests that the LR intervention may have broader benefits beyond improving daily activity performance and reducing stress, although further research is needed to confirm the impact on social relationships.

Similar to the intervention group, the control group also showed an increase in COPM performance scores from a preintervention score of 4.34 ± 1.07 to a postintervention score of 5.62 ± 0.95 (*p* < 0.05). However, unlike the intervention group, the improvement in COPM satisfaction within the control group did not reach statistical significance (*p* > 0.05).

Interestingly, while the control group also exhibited a slight decrease in SRI scores, indicating a potential decrease in stress levels, this change was not statistically significant (*p* > 0.05). This suggests that while the control group may have experienced some natural improvements over time, the LR intervention may offer additional benefits in reducing stress compared to the control group.

Although positive trends were observed in most WHOQOL-BREF quality-of-life categories after the intervention, none reached statistical significance. However, a noteworthy exception was social relationships, which showed a surprising decrease (Table 3). This unexpected finding warrants further investigation to understand its underlying causes and potential implications.

To enhance the assessment of the LR intervention's effectiveness, we conducted between-group comparisons of the change scores in the outcome measures using the Mann–Whitney *U* test. The Stress Response Inventory demonstrated a statistically significant decrease in the intervention group, as illustrated in Table 3.

Although the findings suggest positive trends in several areas, including COPM performance, stress levels (SRI), and certain quality-of-life categories in WHOQOL-BREF, these findings fell short of achieving statistical significance in some instances. This necessitates further investigation and potentially a larger sample size to conclusively determine the full impact of the LR intervention.

In addition to the quantitative measures, the occupational questionnaire provided valuable qualitative insights into the occupational engagement and time usage of participants in the LR intervention group. Analyzing time allocation through the OQ empowered participants to identify areas for improvement and increase their engagement in meaningful activities. This included pursuing hobbies, volunteering, and spending quality time with loved ones. As one participant aptly stated, “The OQ opened my eyes to how I was using my time. With the support of the LR program, I was able to make changes that brought me more joy and fulfillment.”

The OQ also reflected positive shifts in participants' health habits. Sleep patterns, eating habits, and exercise routines all showed improvement. For instance, one participant reported, “Since using the OQ, I've started planning my meals and incorporating more nutritious options. I feel much more energized now and have the stamina to tackle my goals.” These changes, echoed by other participants, contributed to increased energy levels, improved mood, and greater motivation to engage in meaningful activities.

By analyzing both the OQ data and quantitative measures, this study provides a comprehensive understanding of the positive impact of the LR intervention on participants' occupational engagement, time allocation patterns, and overall well-being. As one participant summarized, “Before the LR program, I felt like I was wasting a lot of time. Now, I'm able to replace those activities with ones I truly enjoy.” This suggests that the LR program empowers participants to reclaim control over their time and lead fulfilling lives.

## 4. Discussion

The primary objective of this study was to evaluate the impact of a LR intervention on the occupational participation, satisfaction, perceived stress levels, and quality of life among KUS. The LR intervention, which focused on enhancing occupational performance and well-being, involved a combination of individual and group sessions that guided participants through the process of identifying their occupational goals, establishing healthy routines and habits, and developing strategies for managing their time and energy.

The findings demonstrate promising evidence for the effectiveness of LR in promoting desired outcomes among KUS. The most significant result is the statistically significant improvement in engagement in meaningful activities for the LR group compared to the control. This finding supports the core principles of LR, which emphasize optimizing occupational performance through addressing various aspects of daily life.

While not all comparisons reached statistical significance, several noteworthy trends emerged suggesting a positive impact of LR. The LR group displayed higher COPM satisfaction scores, lower SRI scores (indicating reduced stress), and higher scores across all WHOQOL-BREF categories, suggesting improvements in occupational performance, stress management, and overall quality of life. These promising trends warrant further investigation to confirm with statistically significant findings.

Our findings resonate with a recent scoping review by Hirvonen and Johansson [21], highlighting the positive effects of LR interventions on health-related quality of life, mental well-being, and occupational performance across diverse populations. Their study also revealed qualitative data indicating improvements in self-esteem, new relationship formation, and increased engagement in meaningful activities—a theme consistent with our observations.

Furthermore, LR has been shown to be effective in enhancing physical and mental health, occupational functioning, and life satisfaction in various populations, including the elderly and individuals with chronic conditions [16, 21–23]. Our study further emphasizes the adaptability of LR principles to different populations and settings, as evidenced by the establishment of healthy habits in the LR group of KUS.

The establishment of healthy habits observed in our study holds promise for enhancing the well-being of university students—a demographic confronting unique challenges related to academic pressures, peer influences, and lifestyle adjustments. This reinforces the value of LR as a versatile and effective intervention for promoting holistic well-being and quality of life across diverse populations.

### 4.1. Limitations

While the results of this study offer promising preliminary support for the effectiveness of LR interventions in enhancing various aspects of well-being among KUS, several limitations must be acknowledged. The most notable limitation is the relatively small sample size, which may have affected the statistical significance of certain outcomes. With a larger and more diverse sample, we might have observed statistically significant differences in additional outcome measures, thereby enhancing the robustness of our findings.

The relatively short duration of the LR intervention, spanning 10 weeks, may not have allowed sufficient time for some changes to become statistically significant. Longer-term intervention with follow-up assessments might provide a more comprehensive understanding of the sustained effects of the LR intervention on KUS.

The study's findings may be limited by the fact that it was conducted with a specific demographic of occupational therapy students at one university. The predominance of female participants (>80% in both groups) and the fact that the students all knew each other could have influenced how they interacted within the LR intervention, potentially affecting their experiences and outcomes.

The authors' involvement in both designing and delivering the LR intervention introduces potential bias into the study. Implementing double-blind research designs with independent researchers conducting interventions and assessments can help to mitigate this bias in future studies.

The limitations of this study, including the small sample size, short intervention duration, and specific population, highlight important areas for future research. Addressing these limitations will enhance the external validity of the findings and provide a more comprehensive understanding of the effectiveness of LR interventions for KUS. Specific future research directions include replication with larger and more diverse KUS populations, investigating the optimal duration of LR interventions, comparing LR to other interventions, exploring the effectiveness of LR in specific KUS populations, utilizing double-blind research designs, exploring the long-term effects of LR interventions, and investigating the cost-effectiveness of LR interventions.

By addressing these limitations and pursuing future research directions, we can gain a more comprehensive understanding of the potential benefits of LR interventions for KUS and optimize their implementation to enhance the well-being and success of this diverse student population.

### 4.2. Clinical Implications

The findings of this study have significant implications for occupational therapists working with KUS. While further research is needed, the positive trends observed in occupational participation, satisfaction, stress levels, and quality of life suggest that LR interventions hold promise for enhancing the well-being of this population.

#### 4.2.1. Specific LR Strategies for KUS

Our study identified several LR strategies that appeared particularly beneficial for university students:

- Time management: utilizing tools like planners, scheduling apps, and prioritizing tasks to improve academic performance and reduce stress
- Healthy habits and routines: establishing regular sleep schedules, incorporating physical activity, practicing stress management techniques like mindfulness or meditation, and developing healthy eating habits
- Meaningful activity engagement: assisting students in identifying and prioritizing activities that align with their personal values and interests; encouraging participation in social groups, clubs, or activities related to their interests; and overcoming barriers hindering their engagement in meaningful activities

#### 4.2.2. Tailoring Interventions

Occupational therapists can integrate these core LR strategies into their existing practice and tailor them to address the specific needs of individual KUS. By considering factors such as students' academic schedules, personal preferences, and cultural backgrounds, therapists can develop personalized intervention plans that promote well-being and empower students to navigate the challenges of academic life.

## 5. Conclusion

In conclusion, our study provides evidence of the potential benefits of LR interventions for KUS. While some outcome measures did not reach statistical significance, the presence of positive trends in occupational participation, satisfaction scores, and WHOQOL-BREF scores highlights the promise of LR in improving various aspects of well-being for KUS. These findings underscore the holistic and adaptable nature of LR interventions, offering a valuable tool for improving the well-being of university students facing the challenges of academic life. Looking beyond South Korea, the adaptability of LR principles suggests its potential to benefit diverse student populations across various cultural contexts and settings, for example, by incorporating local cultural beliefs and practices into the intervention design. Future research endeavors can further refine and expand our understanding of the role of LR in promoting the well-being of diverse populations, exploring the optimal intervention duration, comparing LR with other interventions, and investigating the long-term sustainability of its effects. By continuing to explore and refine LR interventions, we can empower university students worldwide to thrive in their academic pursuits and achieve holistic well-being.

## Figures and Tables

**Table 1:** Modules of Lifestyle Redesign for Korean university students.

Intervention module	Description
Occupation-related topics	(i) Providing information about occupation (ii) Importance of participating in meaningful occupation (iii) Discussing and developing intervention plans to achieve individual goals (iv) Discussion on how individual occupational performance is going and what problems they are facing

Time usage	(i) Analyze time usage through OQ and determine what routines or habits individuals have (ii) Discussion on how to develop healthier habits

Healthy lifestyle topics	(i) Developing a list of elements that constitute a healthy lifestyle

Eating routines	(i) Food choices, eating habits, frequency of eating

Physical activity	(i) Physical activity choices, frequency, and intensity of physical activity

Sleep routines	(i) Sleep patterns, sleeping problems, factors that affect sleep

Stress management	(i) Identifying self-stress level, what causes stress, stress management techniques

**Table 2:** General characteristics of the participants.

General characteristics	LR group (*n* = 17)	Control group (*n* = 16)
	*n* (%)	*n* (%)
Gender		
M (*n*)	2 (11.8)	3 (18.8)
F (*n*)	15 (88.2)	13 (81.3)
Age (yrs)	20.10	20.0

**Table 3:** Summary of pre- and postintervention and change scores of outcome measure.

Outcome measures	Group	Preintervention (mean ± SD)	Postintervention (mean ± SD)	Change (mean ± SD)	*t*-value	*p* value	Comp. *p* value
COPM							
Performance	LR	4.70 ± 0.98	7.38 ± 1.23	2.68 ± 1.45	-3.533	0.006^∗^	0.372
	Control	4.34 ± 1.07	5.62 ± 0.95	1.28 ± 1.11	-2.562	0.010^∗^	
Satisfaction	LR	5.97 ± 1.46	7.64 ± 1.61	1.67 ± 1.98	-3.333	0.000^∗^	0.175
	Control	5.96 ± 1.51	6.81 ± 1.30	0.85 ± 1.36	-1.832	0.067	
Stress Response Inventory	LR	67.88 ± 21.29	59.06 ± 20.26	−8.82 ± 23.08	-2.768	0.001^∗^	0.003^∗^
	Control	70.88 ± 21.41	68.69 ± 26.43	−2.19 ± 27.39	-0.595	0.552	
WHOQOL							
Physical health	LR	24.59 ± 3.74	25.76 ± 3.78	1.17 ± 3.57	-2.015	0.044^∗^	0.941
	Control	24.06 ± 3.27	24.88 ± 4.22	0.82 ± 3.72	-0.789	0.430	
Psychological	LR	18.41 ± 4.83	20.82 ± 3.48	2.41 ± 4.61	-3.147	0.002^∗^	0.761
	Control	18.38 ± 3.05	19.06 ± 3.92	0.68 ± 3.64	-1.174	0.240	
Social relationships	LR	10.24 ± 1.52	10.76 ± 1.20	0.52 ± 1.58	-1.357	0.175	0.997
	Control	10.31 ± 1.07	10.06 ± 1.23	−0.25 ± 1.37	-0.731	0.465	
Environment	LR	24.71 ± 3.51	27.41 ± 3.65	2.70 ± 3.77	-3.295	0.001^∗^	0.219
	Control	24.25 ± 4.72	25.19 ± 5.07	0.94 ± 4.98	-1.228	0.219	
Total	LR	78.06 ± 11.05	84.35 ± 9.74	6.29 ± 11.07	-3.529	0.000^∗^	0.170
	Control	76.81 ± 11.17	79.63 ± 12.78	2.82 ± 12.78	-1.371	0.170	

Note. COPM = Canadian Occupational Performance Measure; WHOQOL = World Health Organization Quality of Life Scale Abbreviated Version; SD = standard deviation. ^∗^*p* < 0.05.

## Data Availability

The data supporting this study's findings are available on request from the corresponding author. The data are not publicly available due to privacy or ethical restrictions.

## References

[1] Cho H., Yoo S.-K., Park C. J. (2021). The relationship between stress and life satisfaction of Korean University students: mediational effects of positive affect and self-compassion. *Asia Pacific Education Review*.

[2] Sanci L., Williams I., Russell M. (2022). Towards a health promoting university: descriptive findings on health, wellbeing and academic performance amongst university students in Australia. *BMC Public Health*.

[3] Deshpande A. G., Johnson J. R., Casta A. M., Marien M. S., Reiff M. (2023). The impact of a mindfulness-based stress reduction program on university students’ mental health: a mixed-methods evaluation. *Journal of American College Health*.

[4] Wold C., Hallett J., Crawford G., Chih H. J., Burns S., Jancey J. M. (2021). University student health and wellbeing study: a test-retest reliability study of a web-based survey investigating undergraduate student health and wellbeing. *Health Promotion Journal of Australia*.

[5] Assaf I., Brieteh F., Tfaily M., El-Baida M., Kadry S., Balusamy B. (2019). Students university healthy lifestyle practice: quantitative analysis. *Health Information Science and Systems*.

[6] Sharp J., Theiler S. (2018). A review of psychological distress among university students: pervasiveness, implications and potential points of intervention. *International Journal for the Advancement of Counselling*.

[7] Liao C.-C., Hsu C.-H., Kuo K.-P., Luo Y.-J., Kao C.-C. (2023). Ability of the sport education model to promote healthy lifestyles in university students: a randomized controlled trial. *International Journal of Environmental Research and Public Health*.

[8] Solomou S., Logue J., Reilly S., Perez-Algorta G. (2023). A systematic review of the association of diet quality with the mental health of university students: implications in health education practice. *Health Education Research*.

[9] Lee D. S., Padilla A. M. (2016). Predicting South Korean university students’ happiness through social support and efficacy beliefs. *International Journal for the Advancement of Counselling*.

[10] Sakamaki R., Amamoto R., Mochida Y., Shinfuku N., Toyama K. (2005). A comparative study of food habits and body shape perception of university students in Japan and Korea. *Nutrition Journal*.

[11] Cassidy T. B., Richards L. G., Eakman A. M. (2017). Feasibility of a Lifestyle Redesign®-inspired intervention for well older adults. *The American Journal of Occupational Therapy*.

[12] Clark F., Jackson J., Carlson M. (2012). Effectiveness of a lifestyle intervention in promoting the well-being of independently living older people: results of the well elderly 2 randomised controlled trial. *Journal of Epidemiological Community Health*.

[13] Jackson J., Carlson M., Mandel D., Zemke R., Clark F. (1998). Occupation in Lifestyle Redesign: the well elderly study occupational therapy program. *The American Journal of Occupational Therapy*.

[14] Mandel D. R., Jackson J. M., Zemke R., Nelson L., Clark F. A. (1999). *Lifestyle Redesign: Implementing the Well Elderly Program*.

[15] Clark F. A. (2015). *Lifestyle Redesign: The Intervention Tested in the USC Well Elderly Studies*.

[16] Simon A. U., Collins C. E. (2017). Lifestyle Redesign® for chronic pain management: a retrospective clinical efficacy study. *The American Journal of Occupational Therapy*.

[17] Law M., Baptiste S., Carswell A., McColl M. A., Polatajko H., Pollock N. (2014). *COPM: Canadian Occupational Performance Measure*.

[18] Koh K. B., Park J. K., Kim C. H. (2000). Development of the stress response inventory. *Journal of the Korean Neurological Association*.

[19] Min S. K., Kim K. I., Lee C. I., Jung Y. C., Suh S. Y., Kim D. K. (2002). Development of the Korean versions of WHO quality of life scale and WHOQOL-BREF. *Quality of Life Research*.

[20] Smith N. R., Kielhofner G., Watts J. H. (1986). The relationships between volition, activity pattern, and life satisfaction in the elderly. *The American Journal of Occupational Therapy*.

[21] Hirvonen H., Johansson A. (2023). Lifestyle Redesign® with independent living older adults in countries other than the USA. *Scandinavian Journal of Occupational Therapy*.

[22] Salar S., İlhan E., Bülbül Ö., Ekici G. (2022). The effectiveness of a client-centered lifestyle intervention in women with fibromyalgia syndrome: a quasi-experimental controlled study. *Health Care for Women International*.

[23] Shomer L., Roll S. C. (2022). Lifestyle Redesign® intervention for psychological well-being and function in people with fibromyalgia: a retrospective cohort study. *The American Journal of Occupational Therapy*.

